# Bis(imidazo[4,5-*f*][1,10]phenanthroline)dinitratolead(II)

**DOI:** 10.1107/S1600536808041317

**Published:** 2008-12-13

**Authors:** Chun-Xiang Li, Xiao-Lin Zha, Chun-Bo Liu, Xiu-Ying Li, Guang-Bo Che

**Affiliations:** a School of Chemistry and Chemical Engineering, Jiangsu University, Zhenjiang 212013, People’s Republic of China; b Department of Chemistry, Jilin Normal University, Siping 136000, People’s Republic of China

## Abstract

In the title compound, [Pb(NO_3_)_2_(C_13_H_8_N_4_)_2_], the Pb^II^ atom (site symmetry 2) is hexa­coordinated by four N atoms from two *N*,*N*′-bidentate imidazo[4,5-*f*][1,10]phenanthroline (*L*) ligands and two O atoms from two weakly coordinated nitrate ions [Pb—O = 2.872 (5) Å] in an irregular arrangement, which may be ascribed to the stereochemically active lone pair of electrons on the metal ion. In the crystal, inter­molecular bifurcated N—H⋯(O,O) hydrogen bonds connect the mol­ecules into chains propagating along [100]. Adjacent chains inter­act by strong aromatic π–π stacking inter­actions, with a centroid–centroid distance of 3.483 (2) Å.

## Related literature

For the ligand synthesis, see: Steck & Day (1943 ▶). For background, see: Che *et al.* (2006 ▶, 2008 ▶); Thomas *et al.* (2008 ▶); Xu *et al.* (2008 ▶). 
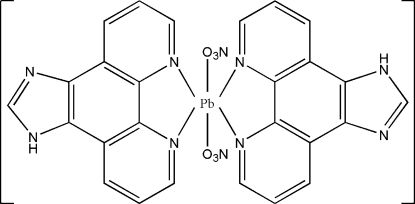


## Experimental

### 

#### Crystal data

[Pb(NO_3_)_2_(C_13_H_8_N_4_)_2_]
*M* *_r_* = 771.68
Monoclinic, 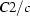

*a* = 19.203 (4) Å
*b* = 7.3948 (15) Å
*c* = 17.392 (4) Å
β = 100.48 (3)°
*V* = 2428.5 (8) Å^3^
*Z* = 4
Mo *K*α radiation
μ = 7.02 mm^−1^
*T* = 292 (2) K
0.56 × 0.22 × 0.11 mm

#### Data collection

Bruker SMART CCD diffractometer
Absorption correction: multi-scan (*SADABS*; Bruker, 2002 ▶) *T*_min_ = 0.165, *T*_max_ = 0.453
10060 measured reflections
2402 independent reflections
2170 reflections with *I* > 2σ(*I*)
*R*_int_ = 0.049

#### Refinement

*R*[*F* ^2^ > 2σ(*F* ^2^)] = 0.026
*wR*(*F* ^2^) = 0.064
*S* = 1.05
2402 reflections
195 parameters
H-atom parameters constrained
Δρ_max_ = 0.89 e Å^−3^
Δρ_min_ = −0.63 e Å^−3^

### 

Data collection: *SMART* (Bruker, 2002 ▶); cell refinement: *SAINT* (Bruker, 2002 ▶); data reduction: *SAINT*; program(s) used to solve structure: *SHELXS97* (Sheldrick, 2008 ▶); program(s) used to refine structure: *SHELXL97* (Sheldrick, 2008 ▶); molecular graphics: *SHELXTL* (Sheldrick, 2008 ▶); software used to prepare material for publication: *SHELXTL*.

## Supplementary Material

Crystal structure: contains datablocks global, I. DOI: 10.1107/S1600536808041317/hb2871sup1.cif

Structure factors: contains datablocks I. DOI: 10.1107/S1600536808041317/hb2871Isup2.hkl

Additional supplementary materials:  crystallographic information; 3D view; checkCIF report

## Figures and Tables

**Table 1:** Selected bond lengths (Å)

Pb—N1	2.540 (3)
Pb—N2	2.606 (3)
Pb—O1	2.872 (5)

**Table 2:** Hydrogen-bond geometry (Å, °)

*D*—H⋯*A*	*D*—H	H⋯*A*	*D*⋯*A*	*D*—H⋯*A*
N3—H3*A*⋯O3^i^	0.86	2.19	3.030 (6)	165
N3—H3*A*⋯O2^i^	0.86	2.35	3.059 (6)	140

Symmetry code: (i) 
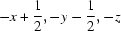
.

**Fig. 1.**
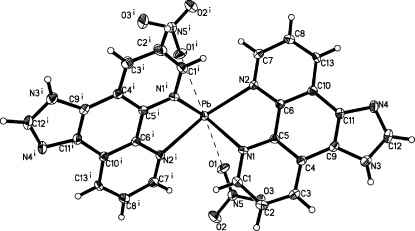
A view of (I). Displacement ellipsoids are drawn at the 30% probability level (arbitrary spheres for the H atoms). Dashed lines indicate semicoordinated bonds [Symmetry code: (i) -x + 1, y, -z + 1/2].

**Fig. 2.**
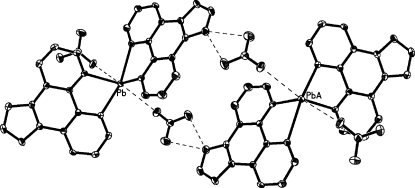
A packing diagram of the title compound, showing a one-dimensional chain-like structure generated by the intermolecular hydrogen bonding. H atoms have been omitted [Symmetry code: (A) -x + 1, -y - 1/2, z - 1/2].

## supplementary crystallographic information

### Comment

1,10-Phenanthroline and its derivatives are very important heteroaromatic N-donor chelating ligands for the construction of coordination compounds. (Che *et al.*, 2008; Xu *et al.*, 2008; Che *et al.*,  2006). Whereas, only a handful of supramolecular architectures based on imidazo[4,5-*f*][1,10]phenanthroline ligand (*L*) have been described (Thomas *et al.*, 2008). The present attempt at synthesizing a new lead complex with *L* ligand gave the title complex, [Pb(C_13_H_8_N_4_)_2_(NO_3_)_2_] (I), whose structure is reported here.

In the title compound I, each Pb atom is hexacoordinated by four N atoms (N1, N1^i^, N2, N2^i^) from two *L* ligand and two O (O1, O1^i^) atoms from two NO_3_^-^ ions (Fig. 1). The Pb—N bond lengths are normal (Table 1), however, O1 and O1^i^ could be described as being semicoordinated with long Pb—O lengths of 2.872 (5) Å. The PbN_4_O_2_ coordination polyhedron approximates a distorted octahedral configuration.

In the crystal structure, the mononuclear complex molecules are linked *via* intermolecular N—H···O and N—H··· N hydrogen bonds (Table 2) forming one-dimensional chains along [100] (Fig. 2). Withal, one-dimensional chains are stabilized by π-π interactions between the *L* rings planes of neighboring molecules with distances of 3.442 Å (Fig. 3), leading to a three-dimensional network.

### Experimental

The *L* ligand was synthesized according to the literature method of Steck & Day (1943). A mixture of Pb(NO_3_)_2_ (0.5 mmol) and the ligand (1 mmol) was dissolved in 15 ml distilled water, then followed by addition of NaOH until the pH value of the system approximately 6.0. Finally the resulting solution was sealed into a 23 ml Teflon-lined stainless steel autoclave and heated at 413 K for 3 d under autogenous pressure. The reaction autoclave slowly cooled to room temperature, yielding yellow block-like crystals of (I) in 62% yield based on Pb.

### Refinement

All H atoms on C atoms were positioned geometrically (C—H = 0.93 Å) and refined as riding, with *U*_iso_(H)= 1.2*U*_eq_(C). The hydrogen atoms of water molecules were located from difference Fourier maps and their positions and *U*_iso_ values were refined freely.

### Crystal data

**Table:**

[Pb(NO_3_)_2_(C_13_H_8_N_4_)_2_]	*F*(000) = 1488
*M**_r_* = 771.68	*D*_x_ = 2.111 Mg m^−^^3^
Monoclinic, *C*2/*c*	Mo *K*α radiation, λ = 0.71073 Å
Hall symbol: -C 2yc	Cell parameters from 3904 reflections
*a* = 19.203 (4) Å	θ = 2.2–26.1°
*b* = 7.3948 (15) Å	µ = 7.02 mm^−^^1^
*c* = 17.392 (4) Å	*T* = 292 K
β = 100.48 (3)°	Block, yellow
*V* = 2428.5 (8) Å^3^	0.56 × 0.22 × 0.11 mm
*Z* = 4	

### Data collection

**Table:**

Bruker SMART CCD diffractometer	2402 independent reflections
Radiation source: fine-focus sealed tube	2170 reflections with *I* > 2σ(*I*)
graphite	*R*_int_ = 0.049
ω scans	θ_max_ = 26.1°, θ_min_ = 2.2°
Absorption correction: multi-scan (*SADABS*; Bruker, 2002)	*h* = −23→23
*T*_min_ = 0.165, *T*_max_ = 0.453	*k* = −9→9
10060 measured reflections	*l* = −20→21

### Refinement

**Table:**

Refinement on *F*^2^	Primary atom site location: structure-invariant direct methods
Least-squares matrix: full	Secondary atom site location: difference Fourier map
*R*[*F*^2^ > 2σ(*F*^2^)] = 0.026	Hydrogen site location: inferred from neighbouring sites
*wR*(*F*^2^) = 0.064	H-atom parameters constrained
*S* = 1.05	*w* = 1/[σ^2^(*F*_o_^2^) + (0.0304*P*)^2^] where *P* = (*F*_o_^2^ + 2*F*_c_^2^)/3
2402 reflections	(Δ/σ)_max_ = 0.005
195 parameters	Δρ_max_ = 0.89 e Å^−^^3^
0 restraints	Δρ_min_ = −0.63 e Å^−^^3^

### Special details

**Table:**

Geometry. All e.s.d.'s (except the e.s.d. in the dihedral angle between two l.s. planes) are estimated using the full covariance matrix. The cell e.s.d.'s are taken into account individually in the estimation of e.s.d.'s in distances, angles and torsion angles; correlations between e.s.d.'s in cell parameters are only used when they are defined by crystal symmetry. An approximate (isotropic) treatment of cell e.s.d.'s is used for estimating e.s.d.'s involving l.s. planes.
Refinement. Refinement of *F*^2^ against ALL reflections. The weighted *R*-factor *wR* and goodness of fit *S* are based on *F*^2^, conventional *R*-factors *R* are based on *F*, with *F* set to zero for negative *F*^2^. The threshold expression of *F*^2^ > σ(*F*^2^) is used only for calculating *R*-factors(gt) *etc*. and is not relevant to the choice of reflections for refinement. *R*-factors based on *F*^2^ are statistically about twice as large as those based on *F*, and *R*- factors based on ALL data will be even larger.

### Fractional atomic coordinates and isotropic or equivalent isotropic displacement parameters (Å^2^)

**Table:**

	*x*	*y*	*z*	*U*_iso_*/*U*_eq_	
C1	0.3676 (2)	−0.2885 (6)	0.1841 (2)	0.0361 (10)	
H1	0.3576	−0.2644	0.2334	0.043*	
C2	0.3181 (3)	−0.3843 (7)	0.1316 (3)	0.0472 (12)	
H2	0.2772	−0.4283	0.1466	0.057*	
C3	0.3303 (2)	−0.4130 (6)	0.0577 (3)	0.0411 (11)	
H3	0.2973	−0.4749	0.0214	0.049*	
C4	0.3927 (2)	−0.3486 (5)	0.0372 (2)	0.0270 (9)	
C5	0.4423 (2)	−0.2606 (5)	0.0951 (2)	0.0242 (8)	
C6	0.5099 (2)	−0.1997 (5)	0.0783 (2)	0.0242 (8)	
C7	0.6190 (2)	−0.0678 (6)	0.1228 (3)	0.0329 (9)	
H7	0.6505	−0.0117	0.1627	0.040*	
C8	0.6394 (2)	−0.0921 (6)	0.0507 (2)	0.0354 (10)	
H8	0.6845	−0.0580	0.0439	0.043*	
C9	0.4103 (2)	−0.3613 (5)	−0.0386 (2)	0.0284 (9)	
C10	0.5268 (2)	−0.2217 (5)	0.0028 (2)	0.0251 (8)	
C11	0.4729 (2)	−0.2999 (5)	−0.0565 (2)	0.0272 (9)	
C12	0.4129 (3)	−0.3952 (6)	−0.1631 (2)	0.0386 (11)	
H12	0.3985	−0.4253	−0.2155	0.046*	
C13	0.5931 (2)	−0.1660 (5)	−0.0100 (2)	0.0296 (9)	
H13	0.6055	−0.1790	−0.0590	0.035*	
N5	0.2959 (2)	0.1066 (5)	0.1716 (2)	0.0387 (9)	
O2	0.2564 (2)	0.0783 (8)	0.2184 (3)	0.0874 (14)	
N1	0.42843 (17)	−0.2294 (4)	0.16763 (18)	0.0268 (7)	
N2	0.55658 (17)	−0.1214 (4)	0.13726 (18)	0.0262 (7)	
N3	0.3723 (2)	−0.4235 (5)	−0.1083 (2)	0.0359 (8)	
H3A	0.3307	−0.4711	−0.1156	0.043*	
N4	0.4751 (2)	−0.3210 (4)	−0.13538 (18)	0.0341 (8)	
O1	0.35914 (17)	0.1449 (5)	0.19411 (19)	0.0508 (9)	
O3	0.2706 (2)	0.0901 (7)	0.1014 (2)	0.0747 (12)	
Pb	0.5000	0.01345 (3)	0.2500	0.02722 (9)	

### Atomic displacement parameters (Å^2^)

**Table:**

	*U*^11^	*U*^22^	*U*^33^	*U*^12^	*U*^13^	*U*^23^
C1	0.034 (3)	0.047 (2)	0.030 (2)	−0.005 (2)	0.013 (2)	0.0003 (19)
C2	0.032 (3)	0.068 (3)	0.044 (3)	−0.018 (2)	0.013 (2)	−0.006 (2)
C3	0.032 (3)	0.055 (3)	0.035 (3)	−0.017 (2)	0.003 (2)	−0.008 (2)
C4	0.024 (2)	0.0303 (19)	0.026 (2)	−0.0021 (16)	0.0025 (17)	0.0008 (16)
C5	0.022 (2)	0.0264 (18)	0.024 (2)	0.0013 (16)	0.0046 (17)	0.0018 (15)
C6	0.024 (2)	0.0247 (18)	0.023 (2)	0.0014 (15)	0.0024 (17)	0.0016 (15)
C7	0.025 (2)	0.041 (2)	0.031 (2)	−0.0063 (19)	0.0020 (19)	−0.0061 (18)
C8	0.027 (3)	0.046 (2)	0.035 (3)	−0.005 (2)	0.010 (2)	0.0001 (19)
C9	0.026 (2)	0.032 (2)	0.025 (2)	−0.0007 (17)	−0.0023 (18)	−0.0004 (16)
C10	0.023 (2)	0.0264 (19)	0.025 (2)	0.0021 (17)	0.0033 (16)	0.0028 (15)
C11	0.032 (2)	0.0283 (19)	0.021 (2)	0.0018 (17)	0.0034 (17)	0.0013 (15)
C12	0.047 (3)	0.043 (2)	0.022 (2)	0.000 (2)	−0.003 (2)	−0.0042 (18)
C13	0.030 (2)	0.041 (2)	0.020 (2)	0.0006 (18)	0.0111 (18)	−0.0019 (16)
N5	0.035 (2)	0.051 (2)	0.030 (2)	0.0030 (18)	0.0059 (18)	−0.0036 (16)
O2	0.048 (3)	0.171 (4)	0.049 (3)	−0.009 (3)	0.024 (2)	0.008 (3)
N1	0.0184 (18)	0.0355 (17)	0.0273 (18)	−0.0007 (14)	0.0065 (14)	0.0000 (13)
N2	0.0235 (19)	0.0331 (17)	0.0217 (17)	−0.0045 (14)	0.0031 (14)	−0.0022 (13)
N3	0.031 (2)	0.0431 (19)	0.032 (2)	−0.0073 (17)	0.0007 (17)	−0.0029 (16)
N4	0.041 (2)	0.0406 (19)	0.0202 (18)	−0.0029 (17)	0.0032 (16)	−0.0022 (14)
O1	0.0265 (19)	0.068 (2)	0.053 (2)	−0.0076 (16)	−0.0049 (16)	−0.0099 (17)
O3	0.054 (3)	0.133 (4)	0.034 (2)	−0.016 (3)	−0.0015 (19)	−0.009 (2)
Pb	0.02505 (15)	0.03545 (14)	0.02129 (13)	0.000	0.00458 (9)	0.000

### Geometric parameters (Å, °)

**Table:**

C1—N1	1.327 (5)	C9—N3	1.376 (5)
C1—C2	1.387 (6)	C10—C13	1.393 (6)
C1—H1	0.9300	C10—C11	1.442 (5)
C2—C3	1.364 (6)	C11—N4	1.390 (5)
C2—H2	0.9300	C12—N4	1.323 (5)
C3—C4	1.396 (6)	C12—N3	1.353 (6)
C3—H3	0.9300	C12—H12	0.9300
C4—C5	1.413 (5)	C13—H13	0.9300
C4—C9	1.423 (5)	N5—O2	1.227 (5)
C5—N1	1.355 (5)	N5—O3	1.235 (5)
C5—C6	1.453 (5)	N5—O1	1.239 (5)
C6—N2	1.362 (5)	Pb—N1	2.540 (3)
C6—C10	1.418 (5)	Pb—N2	2.606 (3)
C7—N2	1.329 (5)	Pb—O1	2.872 (5)
C7—C8	1.392 (6)	N3—H3A	0.8600
C7—H7	0.9300	Pb—N1^i^	2.540 (3)
C8—C13	1.365 (6)	Pb—N2^i^	2.606 (3)
C8—H8	0.9300	O1—Pb^i^	2.872 (5)
C9—C11	1.372 (6)		
			
N1—C1—C2	123.5 (4)	C9—C11—N4	111.8 (4)
N1—C1—H1	118.3	C9—C11—C10	121.0 (3)
C2—C1—H1	118.3	N4—C11—C10	127.1 (4)
C3—C2—C1	119.0 (4)	N4—C12—N3	113.9 (4)
C3—C2—H2	120.5	N4—C12—H12	123.0
C1—C2—H2	120.5	N3—C12—H12	123.0
C2—C3—C4	119.4 (4)	C8—C13—C10	118.7 (4)
C2—C3—H3	120.3	C8—C13—H13	120.6
C4—C3—H3	120.3	C10—C13—H13	120.6
C3—C4—C5	118.3 (4)	O2—N5—O3	117.3 (4)
C3—C4—C9	125.0 (4)	O2—N5—O1	121.2 (4)
C5—C4—C9	116.6 (4)	O3—N5—O1	121.4 (4)
N1—C5—C4	121.4 (3)	C1—N1—C5	118.4 (3)
N1—C5—C6	118.0 (3)	C1—N1—Pb	121.2 (3)
C4—C5—C6	120.7 (3)	C5—N1—Pb	117.9 (2)
N2—C6—C10	121.1 (4)	C7—N2—C6	118.5 (3)
N2—C6—C5	118.0 (3)	C7—N2—Pb	122.8 (3)
C10—C6—C5	120.9 (3)	C6—N2—Pb	115.0 (2)
N2—C7—C8	122.9 (4)	C12—N3—C9	106.7 (4)
N2—C7—H7	118.6	C12—N3—H3A	126.7
C8—C7—H7	118.6	C9—N3—H3A	126.7
C13—C8—C7	119.9 (4)	C12—N4—C11	102.6 (4)
C13—C8—H8	120.1	N1—Pb—N1^i^	90.01 (14)
C7—C8—H8	120.1	N1—Pb—N2^i^	84.09 (10)
C11—C9—N3	105.0 (3)	N1^i^—Pb—N2^i^	64.03 (10)
C11—C9—C4	123.6 (4)	N1—Pb—N2	64.03 (10)
N3—C9—C4	131.4 (4)	N1^i^—Pb—N2	84.09 (10)
C13—C10—C6	118.9 (4)	N2^i^—Pb—N2	135.02 (14)
C13—C10—C11	124.2 (3)	O1—Pb—N1	70.65 (10)
C6—C10—C11	116.9 (4)	O1—Pb—N2	111.75 (10)
			
N1—C1—C2—C3	−3.2 (7)	C11—C10—C13—C8	179.3 (4)
C1—C2—C3—C4	1.2 (7)	C2—C1—N1—C5	1.6 (6)
C2—C3—C4—C5	2.1 (7)	C2—C1—N1—Pb	163.1 (4)
C2—C3—C4—C9	−176.7 (4)	C4—C5—N1—C1	2.0 (5)
C3—C4—C5—N1	−3.8 (6)	C6—C5—N1—C1	−178.4 (3)
C9—C4—C5—N1	175.1 (3)	C4—C5—N1—Pb	−160.2 (3)
C3—C4—C5—C6	176.6 (4)	C6—C5—N1—Pb	19.5 (4)
C9—C4—C5—C6	−4.5 (5)	C8—C7—N2—C6	−1.5 (6)
N1—C5—C6—N2	2.6 (5)	C8—C7—N2—Pb	−158.6 (3)
C4—C5—C6—N2	−177.7 (3)	C10—C6—N2—C7	−0.9 (5)
N1—C5—C6—C10	−177.6 (3)	C5—C6—N2—C7	178.9 (3)
C4—C5—C6—C10	2.0 (5)	C10—C6—N2—Pb	157.9 (3)
N2—C7—C8—C13	3.2 (7)	C5—C6—N2—Pb	−22.3 (4)
C3—C4—C9—C11	−178.6 (4)	N4—C12—N3—C9	0.6 (5)
C5—C4—C9—C11	2.6 (6)	C11—C9—N3—C12	−0.5 (4)
C3—C4—C9—N3	4.8 (7)	C4—C9—N3—C12	176.5 (4)
C5—C4—C9—N3	−173.9 (4)	N3—C12—N4—C11	−0.4 (5)
N2—C6—C10—C13	1.6 (5)	C9—C11—N4—C12	0.1 (4)
C5—C6—C10—C13	−178.1 (3)	C10—C11—N4—C12	−178.9 (4)
N2—C6—C10—C11	−177.7 (3)	C1—N1—Pb—N1^i^	93.6 (3)
C5—C6—C10—C11	2.5 (5)	C5—N1—Pb—N1^i^	−104.8 (3)
N3—C9—C11—N4	0.2 (4)	C1—N1—Pb—N2^i^	29.7 (3)
C4—C9—C11—N4	−177.1 (4)	C5—N1—Pb—N2^i^	−168.7 (3)
N3—C9—C11—C10	179.3 (3)	C1—N1—Pb—N2	177.0 (3)
C4—C9—C11—C10	2.0 (6)	C5—N1—Pb—N2	−21.4 (2)
C13—C10—C11—C9	176.2 (4)	C7—N2—Pb—N1	179.9 (3)
C6—C10—C11—C9	−4.5 (5)	C6—N2—Pb—N1	22.2 (2)
C13—C10—C11—N4	−4.9 (6)	C7—N2—Pb—N1^i^	−87.2 (3)
C6—C10—C11—N4	174.4 (4)	C6—N2—Pb—N1^i^	115.1 (3)
C7—C8—C13—C10	−2.3 (6)	C7—N2—Pb—N2^i^	−130.6 (3)
C6—C10—C13—C8	0.0 (6)	C6—N2—Pb—N2^i^	71.7 (2)

Symmetry codes: (i) −*x*+1, *y*, −*z*+1/2.

### Hydrogen-bond geometry (Å, °)

**Table:**

*D*—H···*A*	*D*—H	H···*A*	*D*···*A*	*D*—H···*A*
N3—H3A···O3^ii^	0.86	2.19	3.030 (6)	165
N3—H3A···O2^ii^	0.86	2.35	3.059 (6)	140

Symmetry codes: (ii) −*x*+1/2, −*y*−1/2, −*z*.
